# Longitudinal trajectories of memory among middle-aged and older people with hearing loss: the influence of cochlear implant use on cognitive functioning

**DOI:** 10.3389/fnagi.2023.1220184

**Published:** 2023-09-14

**Authors:** Christiane Völter, Lisa Götze, Stefan Dazert, Jan Peter Thomas, Stefan Thomas Kamin

**Affiliations:** ^1^Department of Otorhinolaryngology, Head and Neck Surgery, Catholic Hospital Bochum, Ruhr-University, Bochum, Germany; ^2^Department of Otorhinolaryngology, Head and Neck Surgery, St. Johannes Hospital, Dortmund, Germany; ^3^Fraunhofer Institute for Integrated Circuits IIS, Fraunhofer Center for Applied Research on Supply Chain Services SCS, Nuremberg, Germany

**Keywords:** dementia, hearing loss, cochlear implantation, multilevel growth model, cognitive decline

## Abstract

**Introduction:**

Cochlear implants (CI) are the gold standard intervention for severe to profound hearing loss, a known modifiable risk factor for dementia. However, it remains unknown whether CI use might prevent the age-related cognitive decline. Recent studies are encouraging but are limited, mainly by short follow-up periods and, for ethical reasons, lack of appropriate control groups. Further, as age-related cognitive decline is multifaceted and not linear, other statistical approaches have to be evaluated.

**Materials and methods:**

Immediate and delayed recall as measures of cognitive function were assessed in 75 newly implanted CI users (mean age 65.41 years ± 9.19) for up to 5 years (mean 4.5 ± 0.5) of CI use and compared to 8,077 subjects of the same age range from two longitudinal cohort studies, the Health and Retirement Study (HRS) and the English Longitudinal Study of Aging (ELSA). Linear and quadratic changes in cognitive trajectories were analyzed in detail using mixed growth models, considering possible confounders.

**Results:**

For CI users, the linear time slope showed a significant improvement in the specific domains (recall and delayed recall) over time. The quadratic time slope clearly indicated that the predicted change after CI provision followed an inverted U-shape with a predicted decline 2 years after CI provision. In the hearing-impaired group, a significant decline over time was found, with steeper declines early on and the tendency to flatten out in the follow-up.

**Conclusion:**

Cochlear implant use seems to boost cognitive trajectories in the first years after implantation. However, long-term prevention of dementia seems to need far more than restoration of hearing loss.

## Introduction

Dementia is a current health challenge that can only be expected to grow in future (Cantarero-Prieto et al., 2020; The Lancet Regional Health-Europe, 2022). By 2050, the number of people with age-related cognitive impairment is expected to rise up to 152.8 million (Nichols and Vos, 2021). The Lancet Commission recently named 12 potentially modifiable risk factors for dementia, which account for 40% of the worldwide dementia cases (Livingston et al., 2020). One of these 12 risk factors is hearing impairment, which is a very relevant risk factor due to the high prevalence of age-related hearing loss. Therefore, the question arises as to whether treating hearing loss in midlife and old age might delay or even prevent long-term cognitive decline (Dawes, 2019). Cochlear implant (CI) use has been shown to improve hearing ability in individuals with profound to severe hearing loss and may thereby lead to improvements in cognitive functioning. Despite there is a body of research investigating the effects that CI use has on cognitive functioning (Mosnier et al., 2018; Sarant et al., 2019; Huber et al., 2021; Mertens et al., 2021; Herzog et al., 2022; Ohta et al., 2022; Völter et al., 2022a; Dawes and Völter, 2023), these studies demonstrated some methodological limitations. Only a few studies had a follow-up of more than 2 years after implantation (Cosetti et al., 2016; Mosnier et al., 2018; Herzog et al., 2022; Völter et al., 2022b). Further, in the majority of studies, comorbidities which are known to interfere with cognitive function, such as hypertension, obesity, and alcohol or nicotine consumption were not reported (Hagger-Johnson et al., 2013; Dye et al., 2017; Canavan and O’Donnell, 2022).

Another major issue is the inclusion of an adequate control group (Dawes, 2019; Moberly et al., 2019; Huber et al., 2021). As it is unethical to deny a hearing aid or a CI to a person with severe hearing impairment, different control groups have been used in previous studies, such as the inclusion of a waiting group (Jayakody et al., 2017; Mertens et al., 2021) or healthy controls (Huber et al., 2021). The inclusion of healthy controls is challenging, even if these participants might not suffer from the target disease, it is unclear if any other chronic conditions might be present that affect the outcome variable, such as cognitive function. Further, the “supernormal” control group does not represent the general population and differences between the two groups might be overestimated (Marchesini et al., 2017). Additionally, bias cannot be ruled out in volunteers, as they might be more enthusiastic in joining the investigation than the subjects of the intervention group themselves. Further, one must consider that cognitive decline is highly individual and so large control groups covering the high variability in age are needed.

In a previous study from our group, we compared the cognitive performance in 50 CI recipients with data from the Survey of Health, Aging and Retirement in Europe (SHARE), which is a large population-based study (Börsch-Supan et al., 2013), in the same follow-up interval of 5 years. Results showed, that in comparison to the SHARE sample, CI recipients increased their performance in delayed memory and in working memory (Völter et al., 2022b). However, the control group included in this study did not have an objective assessment on auditory abilities; this is a potential limitation because hearing and cognition are closely related (Powell et al., 2021). This association arises from data first published by Rabbitt (1968), who showed that study participants with a simulated hearing loss had poorer word recall results than normal hearing listeners. These findings have been confirmed by numerous researchers (Uhlmann et al., 1989; Lin, 2011; Heywood et al., 2017) and underlined in Loughrey et al.’s (2018) systematic review and meta-analysis, which included more than 36 studies and an estimated 20,264 participants.

Another open question is the shape of cognitive change among CI users and controls. Most studies have explored linear effects of time, assuming that change in cognition is constant and follows a straight line. However, declines in cognitive performance vary across individuals and are not always captured by linear models (Singer et al., 2003; Muniz Terrera et al., 2008). For example, it may be hypothesized that cognitive decline is steeper in the early stages of aging before leveling off in later stages. Moreover, improvements in cognitive functioning among CI users might be more dominant in the first months after implantation, but this improvement may slow down or even reverse in subsequent years. By analyzing the quadratic effects of time on cognition, complexity of the underlying processes that drive cognitive change over time can be captured more accurately.

Therefore, the present study aimed to compare linear and non-linear long-term effects of CI use on recall memory in middle-aged and older adults with up to 5 years of CI experience with the longitudinal data of two large representative studies: the Health and Retirement Study (HRS) (Smith and Smith, 2011) and the English Longitudinal Study of Aging (ELSA) (Steptoe et al., 2013). We focused on recall memory, a key aspect of cognitive aging, since the ability to remember and retrieve new information is a critical indicator of cognitive health and loss of memory is a known early indicator of Alzheimer’s disease (Glisky, 2007; Albert et al., 2013; Boraxbekk et al., 2015; Park et al., 2017).

## Participants and procedures

### Primary data

This study is based on primary and secondary data. The primary data were derived from 75 persons aged ≥50 years who (a) underwent CI implantation between 2016 and 2018, and (b) performed cognitive assessments with a non-auditory based test battery prior to implantation (interval T1) and at 12 months post implantation (T2). 72/75 (96%) of CI recipients also underwent cognitive assessment 24 months after CI (T3); and 50/75 (66.7%) also underwent cognitive assessment at ≥42 months (mean 4.5 years ± 0.5, range 3.5–5.4 years) after cochlear implantation (T4). The resulting primary data sample consisted of 75 persons who provided data across a mean 2.33 observations (±1.06) and had a mean age of 65.41 years (±9.19). All patients of the primary sample were bilaterally hearing-impaired and the mean 4PTA on the better hearing ear was 81.02 (SD 18.33) dB and 100.92 (SD 9.49) dB on the ear to be implanted. Criteria for CI eligibility were based on the ear to be implanted which implies a mean hearing loss threshold (4PTA) of >70 dB and a monosyllabic speech reception score in quiet in the best aided condition of ≤60% at 65 dB according to the German guidelines for cochlear implantation (Arbeitsgemeinschaft der Wissenschaftlichen Medizinischen Fachgesellschaften (AWMF), 2020; Dazert et al., 2020). Mean duration of hearing loss prior to implantation was 25.59 (SD 15.65) years and the mean duration of deafness on the ear to be implanted 15.01 (SD 12.59) years. Most of the study participants were affected by a slowly progressive hearing loss (*n* = 64) and 11 subjects deafened due to a sudden hearing loss. Table 1 provides a description of the sample.

**TABLE 1:** Descriptive statistics data on the cochlear implant recipients and the ELSA and HRS samples.

	Primary data (CI recipients) (*n* = 75)		Secondary data (HRS + ELSA) (*n* = 8,077)	
	**Mean (SD) or %**	**Range**	**Mean (SD) or %**	**Range**
Age (years)	65.41 (9.19)	50–84	67.44 (8.70)	50–98
Female	60%	0–1	54%	0–1
Higher education	15%	0–1	34%	0–1
Smoking	17%	0–1	14%	0–1
Overweight	63%	0–1	75%	0–1
Alcohol consumption	69%	0–1	63%	0–1
Arterial pressure (mmHg)	95.87 (9.61)	73.33–130	93.62 (11.87)	32.77–237.44
Immediate recall	0.00 (1.00)	−1.90 to 1.98	0.00 (1.00)	−2.39 to 2.77
Delayed recall	0.00 (1.00)	−1.43 to 2.29	0.00 (1.00)	−1.27 to 3.14

Recall measures were standardized across all measurements; numbers are reported over all observations for the respective tests. CI, cochlear implant; ELSA, English Longitudinal Study of Aging; HRS, Health and Retirement Study.

### Secondary data

The secondary data comes from two longitudinal cohort studies, the US-based Health and Retirement Study (HRS) and the UK-based English Longitudinal Study of Aging (ELSA). Both studies are administered biannually to older adults (≥50 years) and have an overlap regarding instruments and tests. We selected both studies because they include objective hearing assessments. The current research uses the 2014 (T1) wave of the HRS as a baseline and the 2016 (T2), 2018 (T3), and 2020 (T4) waves as follow-ups. Regarding the ELSA data, we used the 2012 (T1) wave as a baseline and the 2014 (T2), 2016 (T3), and 2018 (T4) waves as follow-ups. We included only participants with full datasets at baseline and who completed the audiometric assessments. 53% of the participants did not suffer from hearing loss, 41% were mildly hearing impaired and 6% were affected by a severe hearing loss. The studies were combined and analyzed together. The resulting secondary data sample consisted of 8,077 participants who provided data across a mean 2.37 observations (±1.10 observations). Their mean age was 67.44 years (±8.70 years). Table 1 provides a description of the sample.

### Measures

#### Audiometric assessment

All participants in the primary data were indicated for a CI, as confirmed by pure-tone thresholds for each ear at 0.25–8 kHz and speech understanding in quiet assessed preoperatively via the Freiburg monosyllabic speech test at 65 dB sound pressure level (SPL). Participants from the HRS and ELSA conducted a hearing test at the second wave (HRS, 2016; ELSA, 2014). In both study cohorts, the Siemens HearCheck screener device was used to produce a fixed series of three high-frequency tones (3 kHz) and three mid-frequency tones (1 kHz), at decreasing intensities (at 55, 35 and 20 dB for 1 kHz; at 75, 55 and 35 dB for 3 kHz). HRS and ELSA participants were categorized as follows using based on the best hearing ear (Ray et al., 2018): severe hearing difficulty (heard 0–2 tones), mild hearing difficulty (heard 3–5 tones), and no hearing difficulty (heard all 6 tones). The Siemens HearCheck is known to provide a good sensitivity (78–92%) and acceptable to good specificity (62–95%) in comparison with pure tone audiometry (Abes et al., 2011).

#### Recall memory

We used recall memory scores as measures of cognitive function in the different study cohorts across all measurement points. The test consisted of recalling a list of ten words. First the complete list was presented once. Then, the participants had to recall the words immediately after the words were presented (immediate recall) and after a delay of approximately 10–20 min (delayed recall). For the primary sample the tests were taken from the ALAcog test battery (Völter et al., 2017, 2018, 2021, 2022b). Recall scores in the HRS and ELSA data ranged between 0–10 for each domain. Recall scores in the primary data were represented as inverse efficiency scores. Therefore, we z-standardized the test scores for each sample and at each measurement.

#### Covariates

Socio-demographic covariates at baseline included chronological age in years, sex (0 = male; 1 = female), and highest educational levels. In the primary data a university entrance diploma (German Abitur) was classified as higher-level education (=1), secondary school diploma or below as lower educational level (=0); in the HRS study highest educational level (=1) was indicated by a college degree or more and (=0) by less; in the ELSA study higher education classified as (=1) reflected education above O-level. Participants with a lower educational background were grouped as (=0).

Regarding health-related covariates, current smoking status (0 = no; 1 = yes), mean arterial pressure (MAP), and overweight (0 = BMI < 24.9; 1 = BMI ≥ 25) as well as alcohol consumption as assessed by self-report were included in the analyses. Alcohol consumption in the primary data was assessed with the question whether the person actually drinks any alcoholic beverages at least once or twice a month (0 = no; 1 = yes) (Steptoe et al., 2013); the HRS assessed the daily drinking behavior over an average week in the last 3 months, which we categorized as “1 = yes” if respondents reported drinking at least once per week; and the ELSA asked how often the subjects had alcoholic drinks during the last 12 months ranging from “not at all” to “almost every day”. We classified alcohol consumption as “1 = yes”, if respondents reported on drinking at least once or twice a month; less frequent drinking was coded as “0 = no”.

### Analytical approach

We estimated random-effect multilevel growth models with measurement occasions (level 1) nested within participants (level 2) to assess the effect of time on change in the cognitive outcomes across the samples. Several models were estimated to answer our research questions: a first model (Model 1) included a linear time slope to model the average linear change for each additional measurement occasion within the respective samples; a second model (Model 2) added a quadratic time × time term to estimate the non-linear relationship between time and cognition; a third model (Model 3) and a fourth model (Model 4) were estimated only with the secondary data and included two-way time × hearing and three-way time × time × hearing interactions to explore whether linear and quadratic change over time in cognitive functioning depended on the participants’ hearing status. All models included the covariates as time-independent variables at level 2. Variables were centered around their respective means to provide a more accurate interpretation of the interaction effects. Missing data on the recall outcomes were handled by multilevel models through full information maximum likelihood (FIML) estimation. This approach allows for unbiased and efficient estimation of model parameters even in the presence of missing data.

## Results

### Linear and quadratic change in primary and secondary data

Table 2 shows the results for the predicted linear (Model 1) and quadratic (Model 2) change in the recall measures across both samples. The reported results are unstandardized parameter estimates.

**TABLE 2:** The predicted linear (Model 1) and quadratic (Model 2) change in the recall measures across both samples.

	Primary data (CI)				Secondary data (HRS + ELSA)			
	**Delayed recall**		**Immediate recall**		**Delayed recall**		**Immediate recall**	
	**Model 1**	**Model 2**	**Model 1**	**Model 2**	**Model 1**	**Model 2**	**Model 1**	**Model 2**
Intercept	-0.66*	-0.77**	-0.77**	-0.86**	-0.15***	-0.14***	-0.18***	-0.18***
Age	-0.03***	-0.03***	-0.02*	-0.02*	-0.03***	-0.03***	-0.03***	-0.03***
Female	0.41**	0.48**	0.58**	0.58**	0.28***	0.28***	0.28***	0.28***
Education	0.49*	0.37	0.84***	0.84***	0.33***	0.32***	0.37***	0.37***
Smoking	-0.26	-0.14	-0.25	-0.25	-0.20***	-0.20***	-0.22***	-0.22***
Overweight	-0.11	-0.05	-0.11	-0.11	-0.02	-0.02	-0.03	-0.03
Alcohol consumption	0.33	0.30	0.36	0.36	0.18***	0.18***	0.22***	0.22***
Mean arterial pressure	0.01	0.01	0.01	0.01	-0.00	-0.00	-0.00	-0.00
Hearing loss					-0.21***	-0.21***	-0.26***	-0.26***
Time	0.11***	0.37***	0.09**	0.35***	-0.07***	-0.12***	-0.06***	-0.06**
Time × Time		-0.09*		-0.09*		0.02***		0.00

*p < 0.05; **p < 0.01; ***p < 0.001.

#### Primary data

Regarding the CI recipients, Model 1 findings indicated that delayed recall was lower as participants age (−0.03), higher for females (0.41) and better educated individuals (0.49). The linear time slope showed a significant increase in this cognitive domain over time (0.11). Model 2 revealed a significant and negative quadratic effect of time (−0.09), indicating that the predicted change after cochlear implementation followed an inverted U-Shape. We observed similar findings regarding immediate recall indicating lower scores for older adults (−0.02) and positive effects for being female (0.58) and having a higher education (0.84). The linear change in immediate recall was significantly positive over time (0.09). The quadratic time slope was negative (−0.09), indicating a concave relation between time and immediate recall.

**FIGURE 1:**
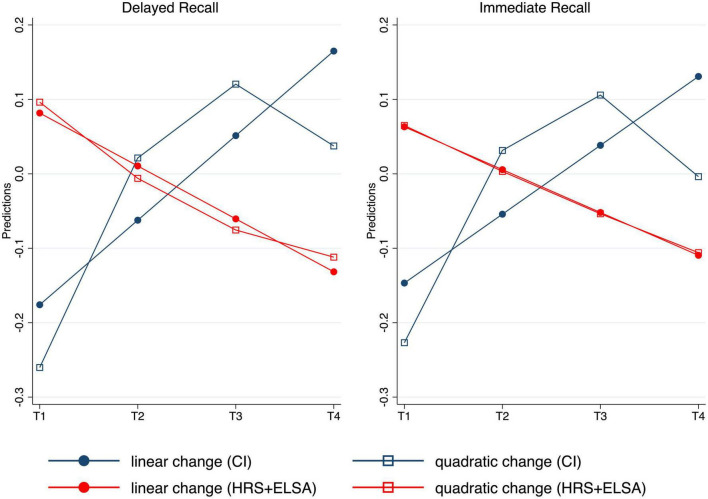
Predicted change in delayed and immediate recall by linear and quadratic time for both samples, adjusted for all covariates. T1 represents baseline data, T2 assessment 2, T3 the third and T4 the fourth measurement.

As can be seen in Figure 1, the predicted non-linear (quadratic) effects provide a more accurate representation of the primary data, indicating cognitive booster effects in the first years after implantation that subsequently leveled out.

#### Secondary data

Regarding the HRS and ELSA participants, Model 1 showed that delayed recall was lower for older adults (−0.03), current smoking behavior (−0.20), and having more objective hearing loss (−0.21). Positive association were found for being female (0.28), higher education (0.33), and alcohol consumption (0.18). The linear time slope indicated a significant decline in this domain over time (−0.07). Model 2 provided a positive and significant effect of quadratic time (0.02). This suggests a convex relation between time and delayed recall, i.e., that declines were steeper early on and tended to flatten out. Again, similar findings were observed for immediate recall indicating lower performance with increasing age (−0.03), smoking (−0.22), and hearing loss (−0.26). Positive associations were found for being female (0.28), higher education (0.37), and alcohol consumption (0.22). The linear time slope was negative (−0.06) indicating declines in immediate recall among the HRS and ELSA participants over time. The quadratic effect of time in Model 2 was not significant.

We performed two supplementary analyses to address methodological concerns. First, we combined the primary and secondary data and estimated all models again to estimate time × sample and time × time × sample interactions. These interactions were significant proving that the reported linear and quadratic trajectories were statistically different between the primary and secondary data (Supplementary Table 1). Secondly, we replicated the analyses for the secondary data with individuals who heard no more than one tone in the best hearing ear to better approximate the threshold for CI implantation. Our findings remained robust indicating negative linear effects of time on both recall measures in the secondary data (Supplementary Table 2).

### Change depending on hearing status in secondary data

**FIGURE 2:**
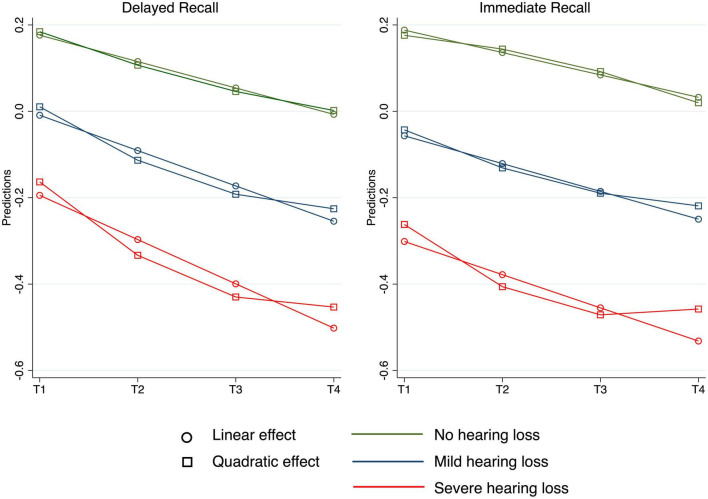
Predicted change in delayed and immediate recall by linear and quadratic time depending on hearing status, adjusted for all covariates. T1 represents baseline data, T2 assessment 2, T3 the third and T4 the fourth measurement.

To further explore the relevance of CI implantation as compared to memory trajectories in the general population, Model 3 and Model 4 were estimated to explore linear and quadratic effects of time and hearing loss in the secondary data. As can be seen in Table 3, the negative linear effect of time (Model 3) in delayed recall was stronger for those with more hearing loss (−0.02). Moreover, Model 4 provided evidence for a significant three-way interaction (0.01) indicating a stronger convex relation between time and hearing loss in delayed recall. Regarding immediate recall, we did not observe a linear time X hearing interaction; however, Model 4 showed that the significant quadratic effect of time was stronger for participants with hearing loss (0.02). Figure 2 illustrates the linear and quadratic trajectories in both cognitive tests for the different hearing groups.

**TABLE 3:** Multilevel growth models predicting memory change in the secondary data depending on hearing status.

	Secondary data (HRS + ELSA)			
	**Delayed recall**		**Immediate recall**	
	**Model 3**	**Model 4**	**Model 3**	**Model 4**
Intercept	-0.16***	-0.17***	-0.18***	-0.19***
Age	-0.03***	-0.03***	-0.03***	-0.03***
Female	0.28***	0.28***	0.28***	0.28***
Education	0.33***	0.33***	0.37***	0.38***
Smoking	-0.20***	-0.20***	-0.23***	-0.23***
Overweight	-0.03	-0.03	-0.03	-0.03
Alcohol consumption	0.18***	0.18***	0.22***	0.22***
Mean arterial pressure	-0.00	-0.00	-0.00	-0.00
Hearing loss	-0.19***	-0.17***	-0.24***	-0.23***
Time	-0.06***	-0.09***	-0.05***	-0.02
Time × Time		0.01		-0.01
Time × Hearing	-0.02**	-0.06**	-0.01	-0.08***
Time × Time × Hearing		0.01*		0.02***

*p < 0.05; **p < 0.01; ***p < 0.001.

## Discussion

The main objective of our study was to investigate the impact of cochlear implant (CI) use on memory function in older adults, specifically focusing on immediate and delayed recall. Our aim was not only to determine whether CI use was associated with changes in memory function, but also to understand how these changes unfolded over time. To achieve this, we employed multilevel models (MLMs). This statistical approach allows for the investigation of changes in outcomes over time, accounting for the nested structure of our data (i.e., multiple observations nested within individuals). MLMs also enable the exploration of both linear (i.e., constant rate of change) and quadratic (i.e., change in the rate of change) time effects. This is particularly important in our study, as we hypothesized that the effects of CI use on memory might not be linear but could potentially show a decelerating or accelerating pattern over time.

We found linear effects of time, indicating significant improvements in both domains. This means that a constant rate of change occurred in the CI users and that the average direction of this change was positive. Including a squared effect of the time variable in the model revealed non-linear patterns of change in recall memory. In contrast to a linear effect, a quadratic effect represents the change in the rate of change over time, which allowed us to prove our hypothesis that the effects of CI use would follow a curved line. This hypothesis is based on the assumption that the use of a CI in older adults triggers cognitive plasticity, characterized by an improvement in memory function due to the restored auditory input. This initial boost is followed by a slower rate of improvement or a leveling-off, representing the engagement of cognitive reserve and the individual’s adaptation to the improved auditory input. Accordingly, findings from the non-linear prediction indicated cognitive improvements after implantation that leveled out subsequently.

In contrast, the combined HRS and ELSA data revealed linear declines in both domains over 6 years as well as a quadratic change in delayed recall. Additional analyses of the observed trajectories were performed depending on the hearing status and showed that individuals with mild or severe hearing loss experienced steeper linear declines in delayed recall. Furthermore, both immediate and delayed recall showed significant declines early in participants with hearing loss, providing evidence for quadratic components of change. These declines decreased and leveled off in the later waves. Participants without hearing loss experienced a steady and consistent decline in cognitive function across both domains.

The findings of the present study make several contributions to the literature. First, the comparison of this particular study population with the combined HRS and ELSA data highlights the significance of our findings in CI users. We discovered anticipated age-related negative trajectories in memory using these secondary data analyses. Additionally, the memory decline of respondents with hearing loss was even more pronounced, as determined by the objective hearing tests. We contend that these results show the positive effects of CI use among older adults by treating secondary data as a rough approximation of a non-experimental comparison group.

Further, there are strong correlations between hearing loss and cognitive decline, as already described by Loughrey et al. (2018). However, most of the studies within Loughrey et al. (2018) are based on cross-sectional data, which makes it difficult to draw conclusive evidence about the long-term relationship between hearing and cognition. Our research used secondary data over 6 years, which also allowed us to investigate how objective hearing loss affected the shape of cognitive decline. For those with mild and severe hearing loss, the time slope showed earlier and more pronounced declines; however, these declines leveled off in later waves according to non-linear modeling of the time slope. This finding is particularly important because it shows how modifiable risk factors influence the heterogeneity of cognitive trajectories later in life.

Our research underlines the importance of considering both linear and non-linear effects of time on cognitive outcomes among CI users. By identifying significant improvements in recall memory over time among such users, the data provide the first evidence for a “booster” effect, meaning that positive change in cognition occurred mostly within the first 12 months of CI use (i.e., from T1 to T2); thereafter, these positive effects decreased and leveled off in later waves, suggesting the potential limits of cognitive plasticity in later life.

These findings are in line with those obtained by early studies of age differences in memory plasticity. For example, Baltes and Kliegl (1992) trained younger and older adults with the Loci Method (Bower, 1970), a classic mnemonic technique for serial learning. Findings based on this training paradigm indicated considerable reserve plasticity in older adults, as indicated by improvements in memory. But, compared with younger adults, there were also clear limits on further improvements.

Similar trends have been reported by intervention studies that have explored long-term effects of cognitive training. For example, the ACTIVE trial reported 10-year effects of intense cognitive training in older adults (Rebok et al., 2014). Post-intervention findings from this study indicated strong and early improvements in memory that slowly leveled off over the observational period. However, it should be noted that only few studies have provided long-term follow-up information (Gross et al., 2012), which indicates the need to further explore longer trajectories of memory training effects over time. Furthermore, it has been observed that trajectories of change may vary depending on the cognitive domain. For instance, findings from the ACTIVE trial indicated that the training effects on reasoning and cognitive speed were sustained for 10 years, while no such sustained effects were observed for the memory outcome.

How to explain these findings? It is possible that other factors, such as the intense training program which usually takes places during the first few years after CI provision and which combines auditory and cognitive elements might have contributed to the observed pattern of cognitive change. Furthermore, one might argue that improvement in speech recognition alone might not be sufficient, and that stimulating and rewarding social environments and social interactions might be crucial to enhance cognition in the long-term follow-up (Anatürk et al., 2021). This fits to studies analyzing the effect of cognitive training interventions and the protective role of social engagement on cognitive functioning and on developing Alzheimer’s disease (Bennett et al., 2006; Kamin et al., 2021).

Considering that hearing loss is associated with a faster cognitive decline, the observation that cognition improves after implantation and that such improvement is maintained until 24 months after CI use is promising. But until now it is not clear whether the close relationship between hearing loss and cognitive decline is causative as research is heterogenous and is based only on a limited number of studies (Loughrey et al., 2018; Livingston et al., 2020; Yeo et al., 2023).

Cognitive functions, especially memory and verbal fluency, have a strong impact on an individual’s ability to communicate effectively. Memory is essential in language comprehension and production, while word-finding abilities play a crucial role in fluent and coherent speech (Akeroyd, 2008). Moreover effective communication is a key component of social activity and engagement. Research has shown that individuals with better communication skills tend to have more active social lives (Pichora-Fuller et al., 2015) as they are more likely to participate in social activities, maintain social relationships, and have a higher quality of life. This is particularly significant in the context of aging, where social participation and interaction can help mitigate feelings of loneliness and isolation, which is an important aspect of healthy aging (Victor and Bowling, 2012). Therefore, potential cognitive improvements following cochlear implantation could lead to enhanced communication skills and increased social activity, thereby improving the overall quality of life for middle-aged and older adults.

Taking this into account, we should encourage older people to have their age-related hearing loss treated. Further research is needed to better understand the underlying mechanisms of cognitive change after cochlear implantation and to identify potential interventions to promote cognitive plasticity in older CI users.

Nevertheless, hearing loss is only one modifiable risk factor for the development of dementia (Sabia et al., 2018; Zaninotto et al., 2018; Livingston et al., 2020; Lövdén et al., 2020). Our results underline the impact of other risk factors such as education and gender on cognitive function, whereas we did not find a negative impact of overweight, smoking or alcohol consumption in both groups. This may be explained by the classification system used or the rather short follow-up of 5 years (Sabia et al., 2018; Livingston et al., 2020).

Our study had several methodological strengths. In contrast to other studies, we controlled for a wide range of potential confounding factors that may affect cognitive decline, such as age, sex, socioeconomic status, blood pressure, obesity, smoking, and alcohol consumption. Controlling for these variables improved the internal validity and provided a more robust examination of the relationship between cognitive functioning and hearing loss among CI users. We also used objective measures of hearing acuity, which is important given that subjective evaluations of hearing loss are often underestimated in people with hearing impairment. Finally, we modeled quadratic effects of time rather than relying on linear predictions. This approach is a considerable strength of our study because it provides a more accurate representation of the complex changes in cognitive outcomes after CI implementation.

## Limitations

While our study provides valuable insights into the trajectory of cognitive functioning following cochlear implantation, it is important to acknowledge its limitations. One such limitation pertains to the covariates included in our analysis as cognitive decline is a complex process and numerous factors influence cognitive trajectories in older adults. Although we have considered a wide range of covariates as highlighted by Livingston et al. (2020), several factors such air-pollution, pre-existing traumatic brain-injury or social isolation were beyond the scope of our data. Furthermore, given the relatively small sample size of our cochlear implant data, we had to be cautious about the risk of overfitting when including too many covariates in our statistical model. Future research with larger sample sizes and more comprehensive data collection could help to address these limitations and provide a more comprehensive understanding of the mechanisms underlying the relationship between CI use and cognitive changes over time.

Furthermore, the two groups differ in the measurement of hearing abilities which limits comparability. Objective measures of hearing function in the ELSA and the HRS study conducted by HearCheck are not as good as the audiometric testing in the CI study, although the accuracy of this screening test battery has been established with a sensitivity of 89% and a specificity of 62% (Abes et al., 2011). In addition, the definition of hearing loss in non-CI studies was slightly different. As cognitive function was measured using auditory-based tests in control studies, the relationship between the trajectories of cognition and hearing may be partially due to verbal assessment. This was not the case in the CI study. Nevertheless, also by using a non-auditory based test in the CI subjects the impact of hearing ability cannot totally be excluded since visual stimuli might also be associated with auditory memory as people tend to automatically name visual stimuli during reading.

Another limitation might be the follow-up time. It usually takes more than 10 years to develop dementia; so our 5-year follow-up time may have been too short to draw robust conclusions. Therefore, longitudinal studies are required. In addition, we compared studies with different observational periods, which could be considered as a methodological limitation regarding temporal coverage and time-historical differences. However, we are confident that incorporating data from studies with varying timeframes helps us to better understand the dynamic and complex processes that develop over different time scales. Moreover, our focus was on exploring average linear and non-linear cognitive changes over longer periods of time rather than investigating a particular timeframe or period of time.

## Conclusion

Overall, our study adds to the growing body of literature suggesting that CI use may have a positive impact on the cognitive functioning of individuals with hearing loss. By identifying non-linear trajectories in cognitive outcomes, our study provides a more nuanced understanding of the complex relationship between cochlear implantation and cognition. These findings have important implications for the development of future interventions aimed at healthy aging among individuals with hearing loss, and provide important guidance for clinicians and researchers working in this field.

## Data availability statement

The original contributions presented in this study are included in the article/Supplementary material, further inquiries can be directed to the corresponding author.

## Ethics statement

Studies involving human participants were reviewed and approved by the Ruhr-University Bochum, Germany (No. 16-5727-BR). The patients/participants provided their written informed consent to participate in this study.

## Author contributions

CV and SK designed the study. LG selected the subjects and collected a part of the data. CV, SK, and LG analyzed and evaluated the data. CV and SK wrote the manuscript, with contributions from LG and critical feedback from SD and JT. All authors contributed to the article and approved the submitted version.
