# Supplementary material for: Longitudinal trajectories of memory among middle-aged and older people with hearing loss: the influence of cochlear implant use on cognitive functioning
Source: Front Aging Neurosci. 2023 Sep 14;15:1220184. doi: 10.3389/fnagi.2023.1220184 (PMC10537213; doi:10.3389/fnagi.2023.1220184)
Supplement: Supplementary file 1 [file Data_Sheet_1.pdf]

Supplementary Table 1: Multilevel growth models predicting memory in the combined data with interaction effects for “sample” (coded as: 0 = secondary data; 1 = primary data; n = 8,152; n = 28,895 observations)

Variables	Delayed recall	Delayed recall	Immediate recall	Immediate recall
Age	-0.147***	-0.148***	-0.107***	-0.107***
Female	1.487***	1.486***	1.338***	1.337***
Education	1.447**	1.447**	1.457***	1.457***
Mean Arterial Pressure	-0.015	-0.015	-0.008	-0.009
Alcohol	0.779	0.785	0.696	0.701
Smoking	-0.898	-0.915	-0.835	-0.835
Obese	-0.577	-0.593	-0.58	-0.59
Time	-0.156*	-0.245	-0.11	-0.11
Sample	-672.257***	-690.363***	-533.89***	-549.58***
Sample × wave	27.854***	86.638***	19.135***	70.040***
wave × wave		0.03		0.01
Sample × wave × wave		-20.977***		-18.164***
Intercept	14.791***	14.955***	12.728***	12.851***

*Notes. *** p<.001, ** p<.01, * p<.05*

Supplementary Table 2: Multilevel growth models predicting memory in the secondary data for individuals who heard 0 or 1 tones in the best hearing ear (n = 99 individuals; n = 340 observations)

Variables	Delayed recall	Immediate recall
Age	-0.072***	-0.068***
	(0.02)	(0.01)
Female	0.348	0.138
	(0.31)	(0.27)
Education	0.753	0.678
	(0.42)	(0.37)
Mean Arterial Pressure	-0.02	0.003
	(0.01)	(0.01)
Alcohol	0.385	0.561*
	(0.32)	(0.27)
Smoking	-0.11	-0.322
	(0.58)	(0.51)
Obese	0.425	0.233
	(0.45)	(0.39)
Time	-0.283***	-0.169*
	(0.08)	(0.07)
Intercept	9.912***	8.917***
	(1.85)	(1.6)
	(0.06)	(0.06)

*Notes: Standard errors are in parentheses. *** p<.001, ** p<.01, * p<.05*
